# Reference Gene Selection for Gene Expression Analysis of Oocytes Collected from Dairy Cattle and Buffaloes during Winter and Summer

**DOI:** 10.1371/journal.pone.0093287

**Published:** 2014-03-27

**Authors:** Carolina Habermann Macabelli, Roberta Machado Ferreira, Lindsay Unno Gimenes, Nelcio Antonio Tonizza de Carvalho, Júlia Gleyci Soares, Henderson Ayres, Márcio Leão Ferraz, Yeda Fumie Watanabe, Osnir Yoshime Watanabe, Juliano Rodrigues Sangalli, Lawrence Charles Smith, Pietro Sampaio Baruselli, Flávio Vieira Meirelles, Marcos Roberto Chiaratti

**Affiliations:** 1 Departamento de Genética e Evolução, Centro de Ciências Biológicas e da Saúde, Universidade Federal de São Carlos, São Carlos, SP, Brazil; 2 Departamento de Medicina Veterinária, Faculdade de Zootecnia e Engenharia de Alimentos, Universidade de São Paulo, Pirassununga, SP, Brazil; 3 Departamento de Reprodução Animal, Faculdade de Medicina Veterinária e Zootecnia, Universidade de São Paulo, São Paulo, SP, Brazil; 4 Departamento de Medicina Veterinária Preventiva e Reprodução Animal, Faculdade de Ciências Agrárias e Veterinárias, Universidade Estadual Paulista, Jaboticabal, SP, Brazil; 5 Unidade de Pesquisa e Desenvolvimento de Registro, Pólo Regional Vale do Ribeira-APTA, Registro, SP, Brazil; 6 MSD Saúde Animal, São Paulo, SP, Brazil; 7 Vida Reprodutiva Consultoria, Cravinhos, SP, Brazil; 8 WTA Tecnologia Aplicada LTDA, Cravinhos, SP, Brazil; 9 Departamento de Cirurgia, Faculdade de Medicina Veterinária e Zootecnia, Universidade de São Paulo, São Paulo, SP, Brazil; 10 Centre de recherche em reproduction animale, Faculté de medicine vétérinaire, Université de Montréal, St. Hyacinthe, QC, Canada; University of Nevada School of Medicine, United States of Amierca

## Abstract

Oocytes from dairy cattle and buffaloes have severely compromised developmental competence during summer. While analysis of gene expression is a powerful technique for understanding the factors affecting developmental hindrance in oocytes, analysis by real-time reverse transcription PCR (RT-PCR) relies on the correct normalization by reference genes showing stable expression. Furthermore, several studies have found that genes commonly used as reference standards do not behave as expected depending on cell type and experimental design. Hence, it is recommended to evaluate expression stability of candidate reference genes for a specific experimental condition before employing them as internal controls. In acknowledgment of the importance of seasonal effects on oocyte gene expression, the aim of this study was to evaluate the stability of expression levels of ten well-known reference genes (*ACTB*, *GAPDH*, *GUSB*, *HIST1H2AG*, *HPRT1*, *PPIA*, *RPL15*, *SDHA*, *TBP* and *YWHAZ*) using oocytes collected from different categories of dairy cattle and buffaloes during winter and summer. A normalization factor was provided for cattle (*RPL15*, *PPIA* and *GUSB*) and buffaloes (*YWHAZ*, *GUSB* and *GAPDH*) based on the expression of the three most stable reference genes in each species. Normalization of non-reference target genes by these reference genes was shown to be considerably different from normalization by less stable reference genes, further highlighting the need for careful selection of internal controls. Therefore, due to the high variability of reference genes among experimental groups, we conclude that data normalized by internal controls can be misleading and should be compared to not normalized data or to data normalized by an external control in order to better interpret the biological relevance of gene expression analysis.

## Introduction

Reproductive performance of bovids has long been recognized as a major contributor to the overall profitability of dairy operations. Heat stress strongly impairs female fertility in Holstein (*Bos taurus*) and buffalo (*Bubalus bubalis*), decreasing the herd reproductive efficiency [1]–[7], and causing a severe economic loss, nearly US$ 1 billion annually [8]. It is a problem that affects about 60% of the world cattle population [1]. Conception rates decline from about 40–60% in cooler months to 10–20% or lower in summer, depending on the severity of the thermal stress [2], [9]–[12]. Several studies have shown that oocytes and embryos at early stages of development are extremely sensitive to heat stress in both species [4], [10], [11], [13]–[21]. Buffaloes are seasonally polyestrous with a marked reduction in their reproductive performance during summer in regions of high latitude [7], [10]–[12], [22], [23]. Therefore, fertility of buffalo females is not only affected by heat stress but also by the sensitivity of these animals to photoperiod. However, the environmental effects on buffalo fertility does not appear to be due to a failure on follicular development, but due to an impairment of oocyte developmental competence [10], [11]. The causes behind this climate-dependent developmental impairment remain widely unknown and studies of the effects of seasonal temperature oscillations on oocyte gene expression are of value in the understanding of the low developmental potential of bovine and buffalo oocytes during summer [14]–[16], [21], [24], [25].

Gene expression analyses rely on the use of internal controls represented by reference genes (also known as housekeeping genes) that are believed to have stable expression patterns, irrespective of experimental treatments [26], [27]. A reference gene is commonly used in gene expression studies to account for inherent variations (e.g. technical and biological) in real-time reverse transcription PCR (RT-PCR) studies [26], [27]. Nonetheless, many experiments are conducted without adequate confirmation that the genes chosen as reference really behave as expected. Indeed, recent reports have indicated that genes usually chosen as internal controls (e.g. *ACTB* and *GAPDH*) in experiments involving somatic cells do not behave as references in experiments using mammalian oocytes and embryos [16], [28]–[31]. This highlights the need for evaluating specific candidates for each experiment, which often requires the selection of three or more reference genes [27]–[32]. For this purpose, the geNorm application can be applied in order to evaluate expression stability and determine genes, among those tested, that should be used [27], [32].

Taking into consideration the importance of seasonal effects on oocyte gene expression, the aim of this work was to evaluate expression stability of ten well-known reference genes (*ACTB*, *GAPDH*, *GUSB*, *HIST1H2AG*, *HPRT1*, *PPIA*, *RPL15*, *SDHA*, *TBP* and *YWHAZ*) using oocytes from different categories of dairy cattle and buffaloes collected during winter and summer. Using the geNorm a normalization factor was provided for cattle (*GUSB*, *PPIA* and *RPL15*) and buffaloes (*YWHAZ*, *GUSB* and *GAPDH*) based on expression of the three most stable reference genes in each species. Additionally, we provide evidence of how selection of internal controls is critical for gene expression analysis by evaluating expression of two non-reference target genes normalized by to reference genes with different expression stability.

## Materials and Methods

All chemicals and reagents used were purchased from Sigma-Aldrich Chemical Co. (St. Louis, MO, USA) unless otherwise stated.

### Ethics statement

The present study was approved by the Bioethics Commission of the Faculdade de Medicina Veterinária e Zootecnia of the Universidade de São Paulo (protocol number 1571/2008), which complies with the ethical principles in animal research. Cattle and buffaloes used in this work were provided by local farms as described below with consent of their owners. No specific permissions were required for their use because it did not involve endangered or protected species. We acknowledge the bovine farms Santa Rita – Agrindus SA (Descalvado, SP, Brazil) and São Jorge (São Pedro, SP, Brazil), and the Unidade de Pesquisa e Desenvolvimento de Registro (buffalo farm), Pólo Regional do Vale do Ribeira-APTA (Registro, SP, Brazil) for supplying animals and management necessary to conduct this work.

### Sample collection

#### Sampling of bovine oocytes

**Figure 1:**
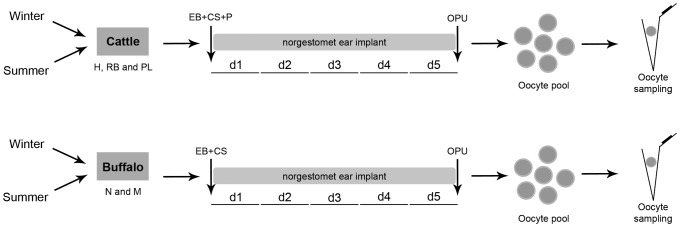
Schematic representation summarizing the experimental model. Three different animal categories of cattle [heifers (H), peak-lactation cows (PL) and repeat-breeder (RB)] and two of buffaloes [nulliparous (N) and multiparous (M)] were used in this study during winter (H, n = 34; PL, n = 32; RB, n = 31; N, n = 9; and M, n = 7) and summer (H, n = 36; PL, n = 37; RB, n = 36; N, n = 8; and M, n = 11). On random days of the estrous cycle (d0), cattle were treated with 2 mg of estradiol benzoate (EB), 150 µg of cloprostenol sodium (CS) and 50 mg of progesterone (P) whereas buffaloes were treated with 2 mg of EB and 530 µg of CS. Furthermore, both cattle and buffaloes received a norgestomet ear implant for five days (until d5) before performing ovum pick up (OPU). Viable cumulus-oocyte complexes (COCs) recovered during OPU sessions were carefully denuded of cumulus cells and the oocytes individually stored for molecular analyses.

All bovine samples used in the present study were collected as reported in a previous work [33]. In brief, Holstein (*B. taurus*) cattle of three different animal categories were analyzed: heifers (H), peak-lactation cows (PL) and repeat-breeder (RB) cows. Heifers were on average 16.8±0.3 months old, cycling, and had never been inseminated; RB were normal cycling lactating cows that had been inseminated several times (range from 4 to 13 services) without becoming pregnant and with no anatomic or infectious abnormality; and PL were normal cycling open cows averaging 110.4±3.8 days in milk. Follicle wave emergence was synchronized using a standard protocol [33] before subjecting cattle to ovum pick up (OPU). The three animal categories were subjected to four OPU sessions (Figure 1), two during winter (H, n = 34; PL, n = 32; and RB, n = 31) and two during summer (H, n = 36; PL, n = 37; and RB, n = 36). However, each animal was subjected only once to OPU. A retrospective analysis was performed to choose the coolest and the warmest periods of the year in the farms where the experiments were conducted [33]. Cumulus-oocyte complexes (COCs) recovered during OPU sessions were morphologically classified based upon ooplasm characteristics and the number of cumulus cell layers as viable or unviable [33]. Approximately 10% of the viable COCs were randomly used for molecular analyses whereas the remaining 90% of the COCs were used for in vitro embryo production, with the results published previously [33]. Oocytes used for molecular analyses were mechanically separated from cumulus cells by vortexing (3 min at maximum speed) followed by three thorough washes in PBS with 0.1% polyvinyl-pyrrolidone (PVP) to completely remove cumulus cells [16], [25], [30], [34]–[38]. Expression of cumulus-specific genes was evaluated in denuded oocytes to validate the protocol used for removal of cumulus cells (File S1). Moreover, before sampling oocytes were thoroughly checked for the presence of somatic cells under a stereomicroscope (Figure S1). Oocytes were individually stored at −80°C in 0.2 ml polystyrene PCR tubes with 1 µl of PBS with 0.1% of PVP and 1 U/µl of RNase inhibitor (RNase OUT, Invitrogen, Carlsbad, CA, USA).

#### Sampling of buffalo oocytes

Buffalo samples used in the present study were collected as described above for cattle, with few modifications. The experiments for collection of these samples were conducted in a farm located in the city of Registro (24°30′07″S 47°50′54″W), São Paulo state in southeast Brazil, during the warm (January; summer) versus cool (July; winter) periods of 2011 and 2012, respectively. A retrospective analysis was also conducted to choose the warmest and the coolest periods of the year in the farm where the experiment was carried out (data not shown). Murrah buffaloes (*B. bubalis*) used in the present work were maintained as described in a previous report [39]. As performed for cattle, two categories of buffaloes were used for oocyte collection: nulliparous (N) and multiparous (M). Nulliparous were normal cycling females with no anatomic or infectious abnormality that averaged 38.9±2.6 months old and never calved; multiparous cows were also cycling, calved on average 3.6±0.6 times and were 92.9±10.6 months old. Multiparous buffalo cows were milked once daily. In order to obtain sufficient oocytes for the purpose of this work, follicle wave emergence was synchronized using a standard protocol before OPU, similarly as performed for cattle [33]. This protocol is based on the use of synthetic hormones that stimulate the synchronized growth of follicles to antral stage within five days of the beginning of the treatment [40]. Briefly, on random days of the estrous cycle (d0), buffaloes were treated with 2 mg of estradiol benzoate (Gonadiol, MSD Saúde Animal, Cotia, SP, Brazil), 530 µg of cloprostenol sodium (Ciosin, MSD Saúde Animal) i.m. plus a norgestomet ear implant (Crestar, MSD Saúde Animal). The norgestomet implant was maintained for five days (until d5) when OPU was performed [40]. The two animal categories were subjected to two OPU sessions (Figure 1), one during winter (N, n = 9; and M, n = 7) and one during summer (N, n = 8; and M, n = 11). Viable COCs recovered during OPU sessions were carefully and thoroughly denuded of cumulus cells and the oocytes stored as described above for bovine samples.

### Quantification of mRNA

#### RNA extraction and reverse transcription

Total RNA was extracted from each individual oocyte using TRIzol reagent (Invitrogen) according to the manufacturer's recommendations, with modifications. In brief, a mix containing 100 µl of TRIzol reagent plus 1 µl of linear acrylamide (5 µg/µl; Ambion Inc., Austin, TX) plus 9 µl of diethylene pyrocarbonate (DEPC)-treated H_2_O (Invitrogen) was added to each sample. Samples were then incubated for 5 min at room temperature before adding 20 µl of chloroform and incubating for 3 min at room temperature. This mixture was centrifuged at 12,000× g for 15 min at 4°C and 50 µl of the supernatant was transferred to a new 0.2 ml polystyrene PCR tube to which 50 µl of isopropanol alcohol was added followed by incubation for 10 min at room temperate and 20 min at −20°C. The samples were centrifuged at 12,000× g for 10 min at 4°C and the supernatant removed with care (it was possible to visualize the pellet of linear acrylamide containing the RNA). Finally, 100 µl of 75% ethanol was added into the tube, centrifuged at 7,500× g for 5 min at 4°C, the supernatant removed (guided again by the pellet of linear acrylamide) and the pellet dried.

The extracted RNA was directly dissolved in 10 µl of DNase I solution (Invitrogen) plus 1 U/µl RNase OUT for DNA degradation as suggested by the manufacturer. To confirm the absence of contaminating DNA, samples were subjected to the amplification protocol with *MT-CO1* primer (see protocol in [41]) before reverse transcription. Over one thousand copies of mitochondrial DNA are present in a single oocyte rendering *MT-CO1* amplification an extremely sensitive method for ascertaining complete digestion of contaminating DNA. Finally, the RNA was immediately reverse transcribed into cDNA using the High-Capacity cDNA Reverse Transcription kit (Applied Biosystems, Foster City, CA, USA) according to manufacturer's protocol. cDNA was stored at −20°C until use.

#### Design of TaqMan assays

A total of ten genes (Table S1), most commonly used as reference in reports of bovine oocytes [16], [27]–[32], [36], [38], [42], were selected for evaluation of bovine and buffalo oocytes. These genes were selected taking into account their roles in the cell (e.g. organization of the cytoskeleton or enzymatic function in energetic metabolism) in an attempt to select genes belonging to distinct functional classes that were not co-regulated. Additionally, two non-reference target genes (Table S1), *HSPA1AB* (which corresponds to amplification of both isoforms *HSPA1A* and *HSPA1B*) and *HSP90AA1* were used to further understand the effect of reference gene selection on final gene expression data. As members of the heat shock protein family, expression of these two target genes is known to be affected by heat stress [25], [43].

Quantification of gene transcripts was carried out using real-time RT-PCR using TaqMan assays. Primers and TaqMan probes were designed using the Primer Express software v.3.1 (Applied Biosystems) based upon sequences available in GenBank (Table S1). Due to unavailability of the selected gene sequences for buffaloes, primers and probes used to amplify transcripts from cattle and buffaloes were based on bovine sequences. PCR products representative of each amplification assay were run onto 2% agarose gels to check the specificity of the amplified fragment. Moreover, to confirm the specificity of primers to buffalo targets, the PCR products were sequenced on an ABI 3730 DNA Analyzer (using the BigDye Terminator v.3.1 Cycle Sequencing kit; Applied Biosystems) and the sequences aligned with the bovine sequences. Whenever possible, primers or probes were designed to anneal on exon-exon junctions and thus avoid genomic DNA amplification. Primers and probes were ordered from Applied Biosystems or Sigma-Aldrich as indicated in Table S1.

#### Preamplification of target cDNAs

Before performing real-time RT-PCR, cDNA was preamplified using the TaqMan PreAmp Master Mix kit (Applied Biosystems) according to manufacturer's recommendations as follows: a 10-µl reaction was prepared containing a pool of all primers used (each one at final concentration of 45 nM) plus 1× TaqMan PreAmp Master Mix plus 4 µl of template (cDNA from samples), subjected to 20 thermal cycles and stored at −20°C. Based on previous tests it was necessary to use 20 cycles of preamplification because of the low starting amount of cDNA regarding the target transcripts (data not published). The linearity of preamplification of all transcripts was determined as suggested by the manufacturer. Briefly, cDNA from pooled samples were subjected to one or two rounds of preamplification (20 thermal cycles each). In the latter case, cDNA from the first round of preamplification was diluted to reach the initial concentration and used as template for the second round of preamplification. Standard curves were generated by real-time RT-PCR (see below) using as template four 5-fold serial-dilutions of preamplified cDNAs from one or two rounds of preamplification. As suggested by the manufacturer, the log of input amount of template versus the delta (Δ) Ct values (Ct is the cycle threshold) for a serial-diluted cDNA was plotted to perform a linear regression. Slope values smaller than 0.1 indicate high amplification efficiency, which supports the use of the 2^−ΔΔCt^ method. Then, the ΔΔCt was calculated to check the linearity of preamplification by comparing the relative amount of a specific transcript after one and two rounds of amplification. As suggested by the manufacturer, we assumed as uniform preamplification ΔΔCt values smaller than ±1.5.

#### Real-time reverse transcription PCR

Real-time RT-PCR was conducted in 15-µl reactions containing 1× TaqMan assay (consisting of 900 nM of primers and 250 nM of probe), 1× TaqMan Gene Expression Master Mix (Applied Biosystems) and 2 µl of template. For each sample, preamplified cDNAs were diluted 125 fold (1 in 125 dilution) to be used as template. Dilution factors were determined based on serial dilutions done to test amplification efficiency (see above). All gene-specific cDNAs preamplified for a particular sample were run in duplicate in the same real-time RT-PCR plate using the ABI PRISM SDS 7500 HT Real-Time PCR System (Applied Biosystems). The following cycling conditions were applied for all real-time RT-PCR reactions: initial denaturation at 95°C for 15 min followed by 40 cycles consisting of 95°C for 15 sec and 60°C for 1 min. Probe fluorescence was read at the end of each extension step (60°C). Since all real-time RT-PCR assays showed high amplification efficiency (see above), target transcript amounts in each sample were linearized according to Livak and Schmittgen [26] by 2^−ΔΔCt^ (normalized values) or 2^−Ct^ (not normalized values). The linearized values from averaged sample duplicates were used to evaluate the expression level of specific genes regarding season and animal category.

### GeNorm analysis

Analysis of gene expression stability was achieved using qBasePLUS software v. 2.3 (Biogazelle, Zwijnaarde, Belgium) based on the geNorm application [32]. This approach relies on the principle that the expression ratio of two perfect reference genes should be identical in all samples, regardless of the experimental condition or cell type [32]. Increasing variation in this ratio corresponds to decreasing stability. By determining the average pair-wise variation between a particular candidate reference gene and all other candidates, the software calculates the gene stability measure (M). Larger M values are associated with greater variations in gene expression [32]. By stepwise exclusion of the least stable gene and recalculation of the M values, the most stable reference genes are identified. Finally, a normalization factor is calculated based on the geometric mean of the expression levels of the best-performing reference genes [32]. To determine the optimal number of reference genes that should be used, the pairwise variation (V) was calculated between two sequential normalization factors (NF): NF_n_ and NF_n+1_. The NF, based on the geometric mean of the expression level of the n best-performing genes, is calculated by stepwise inclusion of an extra, less stable reference gene. When the gene added has a significant effect on the NF, a large variation is verified, which means that the gene should be included as reference [32].

### Statistical analysis

Real-time RT-PCR data were tested for normality of residuals and homogeneity of variances followed by ANOVA using the GLIMMIX procedure of SAS v. 9.3 (SAS/STAT, SAS Institute Inc., Cary, NC) for log normal distribution. The explanatory variables considered for inclusion in the models were animal category, season and interaction of animal category and season. Final results are presented in natural log (Ln) scale (because of the log normal distribution considered) as normalized values of a specific gene transcript by the mean level of the transcript from H (in the case of cattle) or N (in the case of buffaloes) during winter. Downregulation of expression in a specific experimental group may be represented by negative values relative to H or N during winter because of Ln scale. Therefore, to avoid negative values the mean used for data normalization was divided by *e* ^5^, being all data compared against 5, which is the relative mean expression level of H and N during winter. Data are expressed in relation to H or N during winter because these are the category and season with less physiological (lactation) and environmental (heat stress) influence on fertility. Significance was considered at P<0.05. Values are presented as means ± standard error of the mean (S.E.M.).

## Results

### TaqMan assay screening and amplification efficiency

Amplification specificity of all TaqMan assays used was confirmed by running the PCR products on agarose gels or by sequencing the PCR products (in the case of buffalo samples). Only a single assay designed to measure expression of *HIST1H2AG* failed to amplify its target transcript in buffalo samples. With this exception, targets were successfully amplified for all primer pairs listed in Table S1 for both cattle and buffaloes. Moreover, all assays used for gene expression analysis were found to have high efficiency of amplification. Analysis of gene expression used the 2^−ΔΔCt^ method due to the amplification efficiency of target and reference TaqMan assays. Efficiency was approximately equal for all assays tested (slope values of plotting amount of template versus ΔCt values were less than 0.1).

### Evaluation of preamplification uniformity

Single oocytes were used for analysis of gene expression because of the reduced number and variable amount of oocytes available from donors. Samples were subjected to preamplification to increase the starting amount of transcripts of interest from individual oocytes. To check whether preamplification biased the relative amount of target transcripts we compared the relative level of all transcripts after one and two rounds of amplification by calculating the ΔΔCt. As a result all transcripts evaluated, except *HSPA1AB* in cattle, were found to be uniformly amplified. Comparison of the relative level of specific transcripts (by ΔΔCt) after one and two rounds of amplification were on average equal to 0.61±0.14 for bovine samples and 0.24±0.04 for buffalo samples (Table S2). Except for *HSPA1AB* in cattle that was, on average, equal to 2.90±0.43, ΔΔCt values were much lower than the limit 1.5, the criterion suggested by the manufacturer as acceptable for using preamplified cDNA in quantitative PCR. This corroborates the high amplification efficiency of primers used (see above) and justifies the use of preamplification to increase the input amount of cDNA in real-time RT-PCR. In summary, these findings confirm that it is possible to evaluate expression of several genes using single oocytes by preamplifying the cDNAs of interest without biasing the relative amount of a transcript in relation to reference genes.

### Gene expression stability analysis

**Figure 2:**
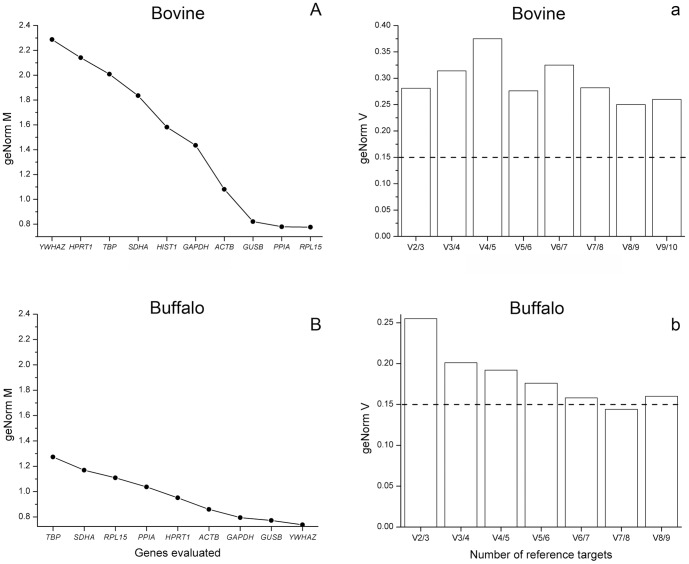
Gene expression stability of the candidate reference genes analyzed by the geNorm application. Average expression stability values (geNorm M) of evaluated genes in bovine (A) and buffalo (B) oocytes, plotted from least stable (left) to most stable (right). Pairwise variation analysis (geNorm V), regarding bovine (a) and buffalo (b) oocytes, between the normalization factor NF_n_ and NF_n+1_, to determine the optimal number of reference genes for normalization. In (a) and (b), the optimal number of reference genes is indicated by values of geNorm V below 0.15 (indicated by the dotted line). In (A), *HIST1* means *HIST1H2AG*.

Gene expression stability in oocytes from cattle and buffaloes was analyzed by the geNorm application (Figure 2). Of ten reference genes evaluated in cattle, *RPL15*, *PPIA* and *GUSB* were found to be the most stable, while, in general, the remaining genes tested were highly variable (Figure 2A). This finding was confirmed by the pairwise variation analysis, as no optimal number of reference genes could be determined in cattle (Figure 2a). The variability between sequential NF (based on the n and n+1 least variable reference targets) was relatively high (geNorm V values lower than 0.15 indicate an optimal NF). This means that based on the geNorm M rank (Figure 2A), even considering nine of ten (V9/10) most stable reference genes in cattle, the geNorm V index remained higher than 0.25 (Figure 2a). According to the geNorm instructions, because no optimal number of reference genes could be determined, it is recommended to use the n-1 reference genes with lowest geNorm M values. This means that for cattle *RPL15*, *PPIA*, *GUSB*, *ACTB*, *GAPDH*, *HIST1H2AG*, *SDHA*, *TBP* and *HPRT1* are the best candidates. With respect to buffaloes, *YWHAZ*, *GUSB* and *GAPDH* were most stably expressed whereas *TBP* was the least stable (Figure 2B). Based on the pairwise variation analysis (Figure 2b), the optimal number of reference genes recommended in this experimental condition is seven. As such, the optimal NF can be calculated as the geometric mean of *YWHAZ*, *GUSB*, *GAPDH*, *ACTB*, *HPRT1*, *PPIA* and *RPL15*. In summary, nine and seven reference genes, respectively, should be used for calculation of a suitable NF for cattle and buffaloes.

### Expression profile of candidate reference genes in bovine and buffalo oocytes

**Figure 3:**
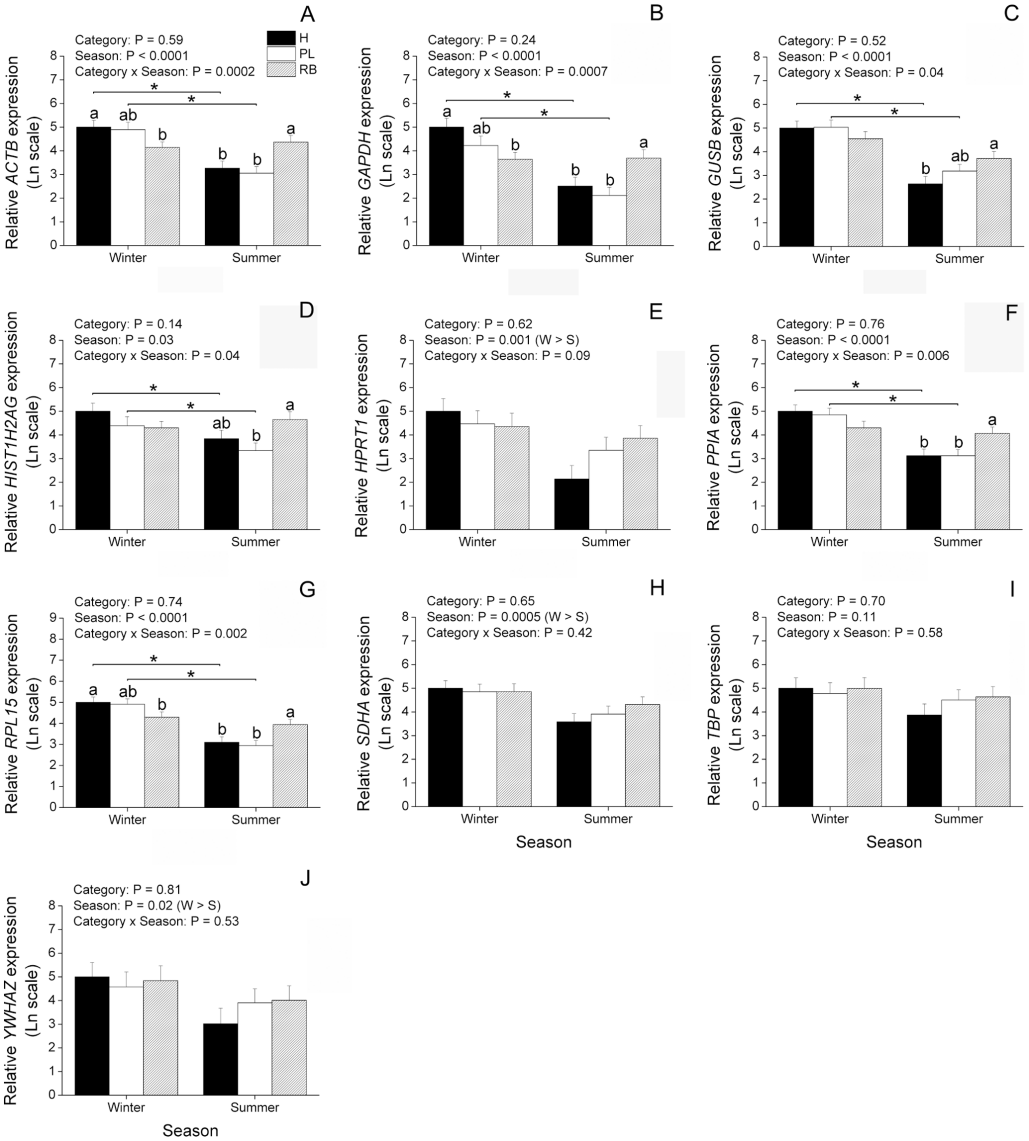
Effect of season on expression of candidate reference genes in immature oocytes from Holstein cattle. Oocytes were retrieved from heifers (H; n = 17 and 15 oocytes, respectively), high-producing cows in peak lactation (PL; n = 16 and 18 oocytes, respectively), and repeat-breeder cows (RB; n = 17 and 17 oocytes, respectively) during winter (W) and summer (S). The amounts of *ACTB* (A), *GAPDH* (B), *GUSB* (C), *HIST1H2AG* (D), *HPRT1* (E), *PPIA* (F), *RPL15* (G), *SDHA* (H), *TBP* (I) and *YWHAZ* (J) transcripts are expressed in relation to H during winter because this was considered as a reference group (see Material and methods for more information). P values for animal category, season and animal category*season are denoted in the insets above each graphic. Different letters over bars denote a significant difference among categories within season (P<0.05). *Difference between seasons within category (P<0.05).

**Figure 4:**
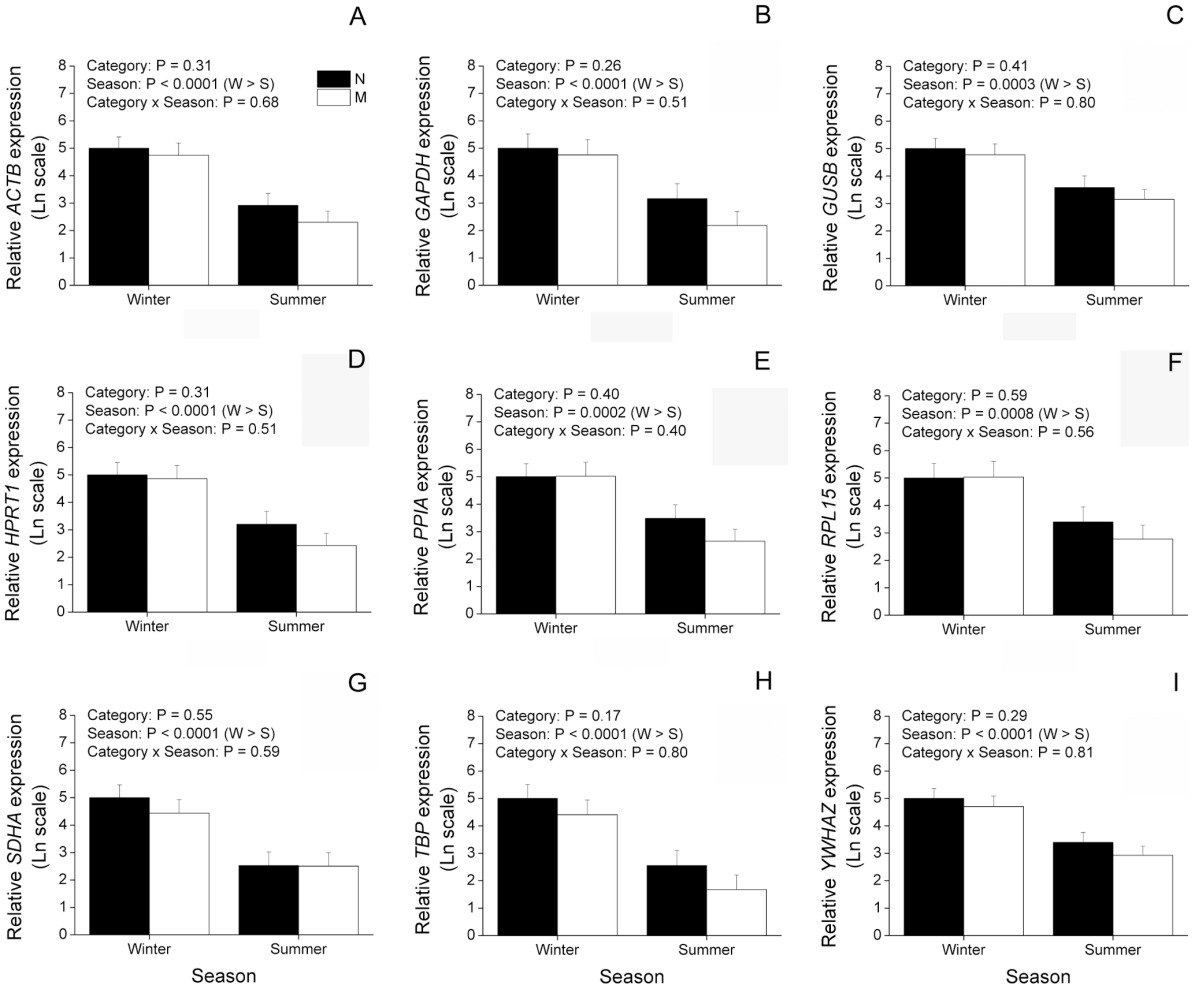
Effect of season on expression of candidate reference genes in immature oocytes from buffaloes. Oocytes were retrieved from nulliparous (N; n = 12 and 11 oocytes, respectively) and multiparous (M; n = 11 and 13 oocytes, respectively) during winter (W) and summer (S). The amounts of *ACTB* (A), *GAPDH* (B), *GUSB* (C), *HPRT1* (D), *PPIA* (E), *RPL15* (F), *SDHA* (G), *TBP* (H) and *YWHAZ* (I) transcripts are expressed in relation to N during winter because this was considered as a reference group (see Material and methods for more information). P values for animal category, season and animal category*season are denoted in the insets above each graphic.

In order to better understand the effect of season and animal category on regulation of reference genes, we investigated their expression levels in the oocytes from cattle and buffaloes. In cattle (Figure 3), most genes (*ACTB*, *GAPDH*, *GUSB*, *HIST1H2AG*, *PPIA* and *RPL15*) showed a significant (P<0.05) interaction of animal category and season, being decreased in summer compared to winter in H and PL, but not in RB (Figure 3). Among these genes, *ACTB*, *GAPDH* and *RPL15* had their expression significantly decreased during winter in RB compared to H (Figure 3). Additionally, expression of *ACTB*, *GAPDH*, *PPIA* and *RPL15* was increased during summer in RB compared to both H and PL. Also with regard to summer, expression of *GUSB* was increased in RB compared to H whereas expression of *HIST1H2AG* was increased in RB compared to PL (Figure 3). With respect to the remaining genes evaluated in cattle (Figure 3), expression of *HPRT1*, *SDHA* and *YWHAZ* was decreased (P<0.05) in summer compared to winter, regardless of animal category, whereas expression of *TBP* did not vary as a result of animal category and season. With respect to buffaloes (Figure 4), expression of all genes was significantly decreased during summer compared to winter (P<0.05), but no effect of animal category or interaction was found. In summary, although these results give evidence of an overall downregulation of expression during summer, differences in the pattern of expression were found among the reference genes evaluated in cattle.

### Effect of reference gene selection on expression values of non-reference target genes in bovine and buffalo oocytes

**Figure 5:**
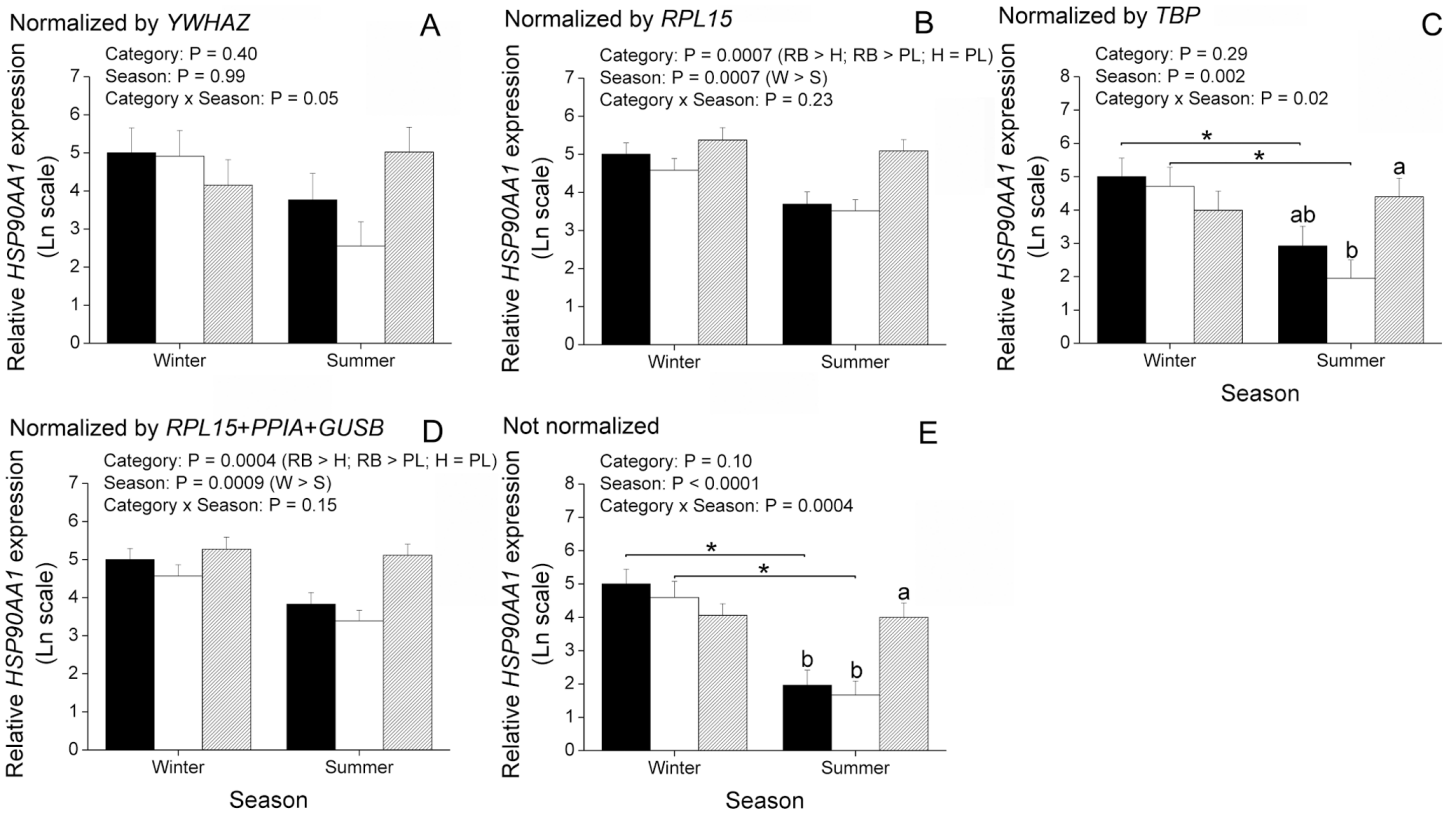
Effect of reference gene selection on expression values of non-reference target genes in immature oocytes from Holstein cattle. Oocytes were retrieved from heifers (H; n = 17 and 15 oocytes, respectively), high-producing cows in peak lactation (PL; n = 16 and 18 oocytes, respectively), and repeat-breeder cows (RB; n = 17 and 17 oocytes, respectively) during winter (W) and summer (S). The amounts of *HSP90AA1* (A–E) transcripts are expressed in relation to H during winter because this was considered as a reference group (see Material and methods for more information). Expression values were normalized either by *YWHAZ* (A), *RPL15* (B), *TPB* (C), geometric mean of *RPL15*, *PPIA* and *GUSB* (D) or not normalized (E). P values for animal category, season and animal category*season are denoted in the insets above each graphic. Different letters over bars denote a significant difference among categories within season (P<0.05). *Difference between seasons within category (P<0.05).

**Figure 6:**
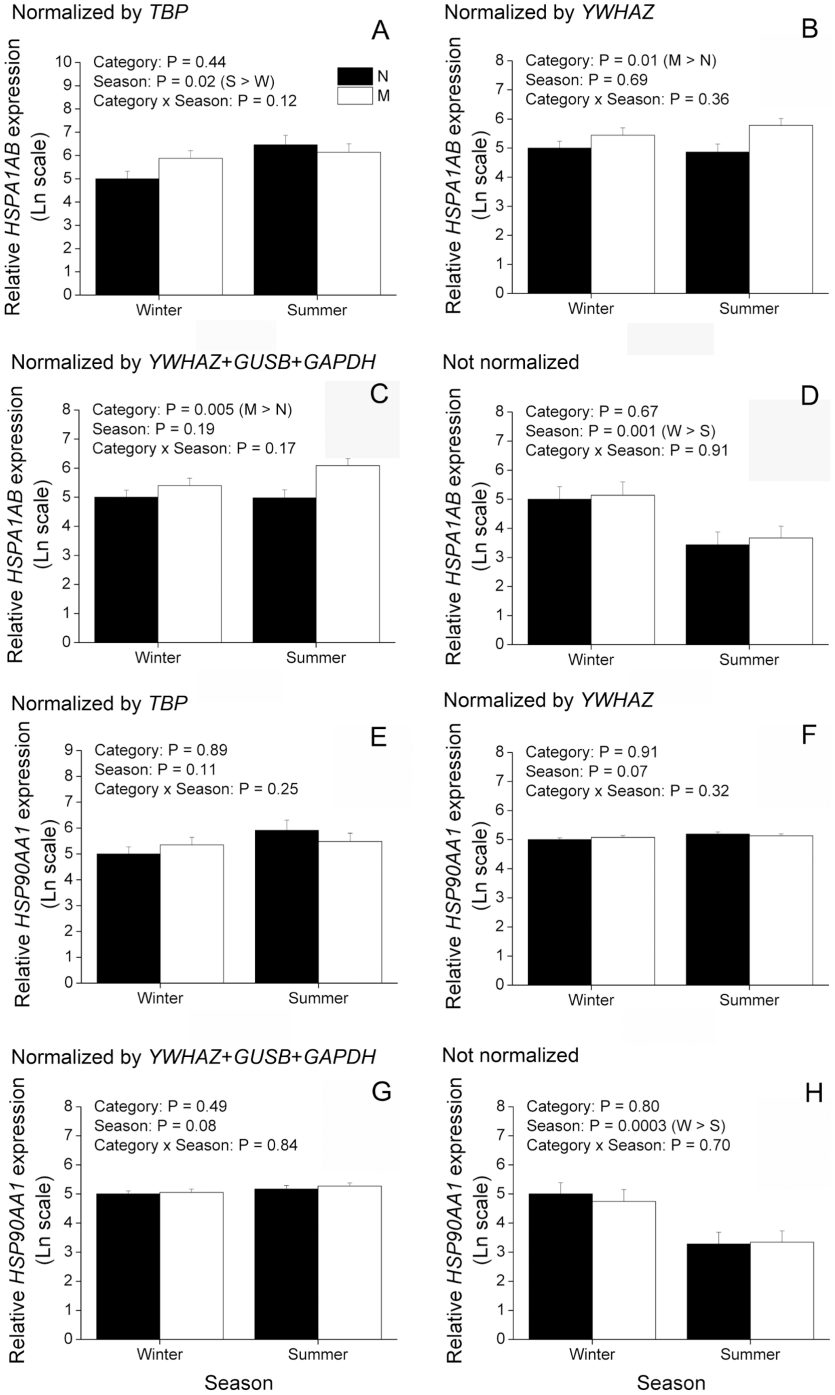
Effect of reference gene selection on expression values of non-reference target genes in immature oocytes from buffaloes. Oocytes were retrieved from nulliparous (N; n = 12 and 11 oocytes, respectively) and multiparous (M; n = 11 and 13 oocytes, respectively) during winter (W) and summer (S). The amounts of *HSPA1A* plus *HSPA1B* (termed in the current work as *HSPA1AB*; A–D) and *HSP90AA1* (E–H) transcripts are expressed in relation to N during winter because this was considered as a reference group (see Material and methods for more information). Expression values were normalized either by *TBP* (A and E), *YWHAZ* (B and F), geometric mean of *YWHAZ*, *GUSB* and *GAPDH* (C and G) or not normalized (D and H). P values for animal category, season and animal category*season are denoted in the insets above each graphic.

Aiming to understand the relevance of reference gene selection for gene expression experiments, we evaluated expression of two non-reference target genes (*HSPA1AB* and *HSP90AA1*) and normalized them to different NFs. With regard to cattle, information from *HSPA1AB* was excluded from analysis because we failed to validate it with our preamplification protocol. A second gene, *HSP90AA1* (Figure 5), displayed an effect (P<0.05) of animal category and season, but not of interaction, when normalized by *RPL15* (the most stable reference gene) or by the geometric mean of the three most stable reference genes (*RPL15*, *PPIA* and *GUSB*). It can be inferred from these data that expression of *HSP90AA1* in cattle drops from winter to summer regardless of animal category (P<0.05). Moreover, regardless of season, expression of *HSP90AA1* is significantly (P<0.05) greater in RB than H or PL, whereas H and PL did not differ from each other. These results are in disagreement with those found for *HSP90AA1* normalized by *YWHAZ* (the least stable reference gene) as no significant effect was detected in this case (Figure 5). Normalization by *TBP*, the only reference gene that did not vary among experimental groups (Figure 3), or the absence of normalization showed a significant (P<0.05) effect of interaction between animal category and season (Figure 5). This latter result indicates that expression of *HSP90AA1* is decreased (P<0.05) in summer when compared to winter in H and PL, but not in RB. Moreover, whereas normalization by *TBP* resulted in greater (P<0.05) levels of *HSP90AA1* during summer in RB when compared to PL, the absence of normalization resulted in greater (P<0.05) levels of *HSP90AA1* during summer in RB when compared to both PL and H (Figure 5). In buffaloes, normalization of *HSPA1AB* and *HSP90AA1* by the most stable reference gene (*YWHAZ*) or by the geometric mean of the three most stable reference genes (*YWHAZ*, *GUSB* and *GAPDH*) yielded similar results (Figure 6). Expression of *HSPA1AB* was significantly (P<0.05) greater in M than N, regardless of season, whereas expression of *HSP90AA1* was not affected by animal category, season or the interaction of them. On the other hand, normalization by the least stable reference gene, *TBP*, resulted in greater (P<0.05) expression of *HSPA1AB* in summer than winter, whereas no effect of animal category, season or interaction was found on *HSP90AA1* when normalized by *TBP* (Figure 6). The absence of normalization for both *HSPA1AB* and *HSP90AA1* resulted in an effect (P<0.05) of season, with both decreased in summer compared to winter (Figure 6). In summary, these results provide clear evidence that reference gene selection has a major effect on expression values of target genes.

## Discussion

The use of real-time RT-PCR for analysis of oocyte gene expression is a powerful tool for evaluating the cytoplasmic transcripts that may influence developmental competence. When considering the effect of season on oocyte viability this tool has been extensively employed in cattle and to a lesser extent in buffaloes [4], [13], [15], [16], [21], [24], [25]. Many of these studies in dairy cattle and buffaloes identified altered patterns of gene expression in oocytes exposed to heat stress that might be associated with their lower developmental competence. Studies of gene expression are also valuable for understanding the effect of photoperiod on buffalo oocytes [10], [11], [21]. However, analysis of gene expression depends on the correct selection of internal controls, as the use of highly variable reference genes may completely alter final gene expression data. Thus, herein we evaluated expression stability of ten well-known reference genes using oocytes isolated from different categories of dairy cattle and buffaloes during winter and summer.

Analysis of gene expression by real-time RT-PCR is the method of choice for studying cytoplasmic factors affecting oocyte competence [4], [13], [15], [16], [21], [24], [25], [43]. Real-time RT-PCR is a very sensitive technique able to detect as little as a single copy of a transcript [44]. However, most methods available for performing real-time RT-PCR were developed for analysis of gene expression using samples recovered from a large number of somatic cells, in which availability of biological material from which to extract RNA is not a major problem. In contrast, those working with oocytes and embryos usually have to deal with a limited amount of samples with minute amounts of RNA. As a result, real-time RT-PCR studies using oocytes usually do not cover many genes at a time and the quality of the results is low. Moreover, these studies usually cannot make use of several biological replicates due to the need for pooling samples (10–20 oocytes) to obtain reliable results [4], [13], [15], [16], [21], [24], [28]–[31], [43]. An alternative to these problems is to preamplify target cDNA before carrying out real-time RT-PCR. Only a few studies have employed preamplification to analyze gene expression in oocytes or embryos [37], [41], [45]–[49], mostly because of the general belief that this method biases the final results. However, if properly validated, preamplification is a valuable tool allowing reliable evaluation of several genes from single cells (e.g. an oocyte) [50]–[52]. Herein we used single oocytes because of the low number of oocytes recovered from individual donors. Moreover, because data generated by real-time RT-PCR are usually highly variable, the use of single oocytes enabled us to analyze between 10 and 20 samples per experimental group and thereby minimize the effect of this variation on final results. We show that preamplification can be accomplished using single oocytes from cattle and buffaloes without interfering with the relative amount of most transcripts. All transcripts, except *HSPA1AB* in cattle, were uniformly preamplified as the ratio between a specific gene and all remaining genes did not change after one and two rounds of preamplification. Thus, real-time RT-PCR could be performed with a sufficient amount of preamplified cDNA, enabling quantification of poorly expressed genes. According to the manufacturer, preamplification enables analysis of up to 100 genes using 1–250 ng of cDNA from single cells. This expands the possibilities of gene expression analysis using single oocytes or embryos allowing for a better understanding of these cells without the need for pooling samples.

Gene expression analyses are dependent on the use of internal controls to normalize for the number of cells used for RNA extraction, as well as taking into account degradation/loss during storage and extraction [27], [32], [53]. It is necessary to establish expression stability of candidate reference genes in all experimental situations because internal controls are expected to express stably regardless of experimental conditions [27], [32], [53]. Expression stability can be checked, for instance, using the geNorm application based on the principle that the expression ratio of two perfect reference genes should be identical in all samples [27], [32]. When considering internal controls for analysis of gene expression in oocytes and embryos several candidate genes should be tested, as expression of common reference genes can be highly variable in these samples [28]–[31], [54]. Moreover, considering the sharp effect of season on oocyte gene expression [14]–[16], [21], [24], [55], [56], the use of multiple reference genes is recommended to measure expression levels accurately. The optimal number of reference genes in any specific experimental situation can also be calculated by the geNorm analysis [32]. Herein we studied ten well-known reference genes (*ACTB*, *GAPDH*, *GUSB*, *HIST1H2AG*, *HPRT1*, *PPIA*, *RPL15*, *SDHA*, *TBP* and *YWHAZ*) [27]–[32] in oocytes retrieved from different categories of dairy cattle and buffaloes during winter and summer. Based on the geNorm analysis, *RPL15*, *PPIA* and *GUSB* were the three genes most stable in cattle whereas *YWHAZ*, *GUSB* and *GAPDH* were the three most stable in buffaloes. Curiously, *YWHAZ* was the most stable gene in buffaloes and the least stable in cattle. In general, the genes evaluated in this study were more stable in buffaloes than cattle. This is confirmed by the finding that no optimal number of reference genes was determined by the geNorm analysis in cattle whereas an optimal number of reference genes could be determined in buffaloes. According to this analysis, gene expression data from buffalo oocytes should be normalized by the geometric mean of *YWHAZ*, *GUSB*, *GAPDH*, *ACTB*, *HPRT1*, *PPIA* and *RPL15*. With respect to cattle, although no optimal number of reference genes was determined, according to the geNorm instructions the NF might be calculated by the geometric mean of nine out of ten most stable genes (*RPL15*, *PPIA*, *GUSB*, *ACTB*, *GAPDH*, *HIST1H2AG*, *SDHA*, *TBP* and *HPRT1*). The need for using as many as seven (buffaloes) or nine (cattle) reference genes for calculation of the NF gives further evidence towards the high variation of reference genes in these oocytes. In summary, these results highlight the importance of selecting appropriate internal controls for analysis of oocyte gene expression.

In an attempt to compare our results with those available in the literature we searched for articles that similarly evaluated expression stability of candidate reference genes with regard to the effect of season or heat stress on oocyte gene expression. Gendelman et al. [16], evaluated expression of two genes, *GAPDH* and *YWHAZ*, when comparing the effect of hot and cold seasons on oocyte and embryo gene expression, in cattle. They found stable expression levels of both genes in all stages investigated, except at the two-cell stage in which expression of *YWHAZ* was reported to be variable. These results are in sharp contrast with ours as *YWHAZ* was the least stable gene according to our analysis using bovine oocytes. Moreover, although *GAPDH* was ranked as the fifth most stable reference gene in our analysis, it was not very stable based on the geNorm M index. This result raises concern as *GAPDH* has been often used as a reference gene in experiments involving the effect of heat stress on oocyte and embryo gene expression in cattle and buffaloes [14], [21], [25], [55]. Based on our results, nine and seven reference genes should be used in order to calculate a NF for bovine and buffalo oocytes collected during winter and summer. However, analysis of so many genes may be impractical depending on experimental conditions and goals. Thus, Vandesompele et al. [32] suggested the minimum use of the three most stable reference genes for analysis of gene expression. Taking this into consideration and based on our results, the NF might be calculated by the geometric average of *RPL15*, *PPIA* and *GUSB* for cattle and *YWHAZ*, *GUSB* and *GAPDH* for buffaloes. Although the use of these three genes for each species does not meet the geNorm recommendations (geNorm V value should be below 0.15), their use represent a trade-off between practical considerations and accuracy as previously suggested [29], [32], [54], [57]. Moreover, taking into account the threshold recognized in software instructions (average geNorm M≤1.0), these three genes chosen for each species were indeed very stable as they were all below the threshold. Therefore, we suggest calculating the NF based on geometric average of these three genes for analysis of oocyte gene expression from dairy cattle and buffaloes during winter and summer.

Normalization by reference genes is widely employed to account for cell number variation in gene expression studies using somatic cells [27], [32] where the number of cells in different samples can vary considerably. However, when using precise cell numbers (e.g. with oocytes that can be individually manipulated), there is lesser need for an internal control [53]. Instead, normalization can be done by the use of an external control (e.g. armored RNA or spikes) added during sampling [31], [35], [45], [53], [58]. An external control can account for RNA degradation and/or loss during sample storage and RNA extraction. Alternatively, if RNA degradation and loss can be minimized, even the external control might be omitted [41]. This represents a significant economy in terms of cost and time for analysis of gene expression, as three or more reference genes are usually employed in most experiments [32]. Furthermore, data that are not normalized by internal controls are more informative as it is possible to visualize the effect of the treatment on gene expression regardless of reference genes. This alternative is especially relevant when gene expression is sharply affected by treatments (e.g. effect of season). In this case, to avoid bias due to uneven response of internal controls to treatments, gene expression can be alternatively evaluated without normalization by reference genes. Herein we evaluated expression levels of reference genes without normalization by internal controls. Based on this analysis it was found an overall downregulation of expression from winter to summer in oocytes from cattle and buffaloes. However, although the genes evaluated were well-known reference genes, they were differentially regulated depending on experimental groups. Among those evaluated in cattle, only *TBP* showed stable expression among experimental groups, *HPRT1*, *SDHA* and *YWHAZ* had their expression affected exclusively by the season and *ACTB*, *GAPDH*, *GUSB*, *HIST1H2AG*, *PPIA* and *RPL15* were affected by an interaction of season and animal category. Even these latter genes showed different patterns of expression from each other, further highlighting an uneven regulation of reference genes in oocytes from cattle. In buffaloes, a more similar pattern of regulation was found among reference genes as the nine genes evaluated were expressed in similar levels within treatments. This means that for cattle the NF based on the geNorm analysis takes into consideration nine genes that expressed differentially depending on treatment. On the other hand, when considering the three most stable genes for calculation of the NF, the similar pattern of expression verified among them supports their use as internal controls. Thus, due to the uneven regulation of reference genes among experimental groups, data normalized by internal controls can be misleading and should be compared to not normalized data or to data normalized by an external control to better interpret the biological relevance of gene expression analysis.

Taking into account expression variability of internal controls, we evaluated expression of two non-reference target genes normalized by reference genes unevenly regulated to further clarify the importance of reference gene selection. For this purpose, *HSPA1AB* and *HSP90AA1* were chosen as target genes as they are known to express differentially as a consequence of heat stress [25], [43]. As a result, we found that different NFs can considerably alter expression results of *HSPA1AB* and *HSP90AA1*. For instance, normalization of *HSP90AA1* in cattle by the least (*YWHAZ*) or the most (*RPL15*) stable reference genes resulted in divergent results. If *YWHAZ* had been chosen as the NF, one would have concluded that expression of *HSP90AA1* is not affected by animal category, season or interaction of these factors. Nonetheless, based on the geNorm analysis *YWHAZ* is not recommended as a NF as it is highly variable. As discussed previously, only an effect of season was verified for *YWHAZ* when it was evaluated without normalization by internal controls. On the other hand, a significant interaction of animal category and season was found for *RPL15*, which may explain the differences found for *HSP90AA1* when normalized by these two reference genes. Normalization by the most stable reference gene, *RPL15*, or by the geometric mean of the three most stable reference genes showed that expression of *HSP90AA1* is actually significantly affected by animal category and season. According to these data, expression of *HSP90AA1* drops from winter to summer regardless of animal category. Moreover, expression of *HSP90AA1* is significantly greater in RB oocytes than H or PL. Yet, normalization by *TBP* or the absence of normalization by internal controls indicates a significant interaction of animal category and season, being expression of *HSP90AA1* lower in oocytes from H and PL during summer than winter, but not in RB. This can be explained by the finding that all internal controls evaluated, except *TBP*, have their expression affected by animal category and season. Thus, normalization by *TBP* or the absence of normalization highlight the overall drop in gene expression found in oocytes from winter to summer. On the other hand, normalization by *TBP* differed from data not normalized with regard to the increase of *HSP90AA1* during summer in RB when compared to H and PL, indicating that although statistically stable, *TPB* might have varied among experimental groups. With respect to buffaloes, normalization of *HSP90AA1* by the least stable (*TBP*), by the most stable (*YWHAZ*) or by the geometric mean of the three most stable reference genes resulted in no statistical difference. This is in agreement with our previous conclusion that reference genes behaved similarly in buffaloes when analyzed without normalization. Moreover, compared to cattle, the geNorm analysis indicated that reference genes are less variable in buffaloes. In spite of this, a difference was noted when *HSPA1AB* was normalized by *TBP* or *YWHAZ*. Normalization by *TBP* highlights an effect of season where *HSPA1AB* is increased in summer compared to winter. However, when normalized by *YWHAZ* this effect is no longer apparent, while in its place, an effect of animal category becomes significant. This latter result indicates that *HSPA1AB* is increased in M compared to N, regardless of season, which is also seen when *HSPA1AB* is normalized by the geometric mean of the three most stable reference genes. These results suggest that although internal controls are more stably expressed in buffaloes, an effect of the NF can be observed on expression of *HSPA1AB*. With respect to expression of *HSPA1AB* and *HSP90AA1* not normalized by internal controls, as seen for reference genes, both target genes were decreased in summer compared to winter regardless of animal category. In summary, these results give clear evidence of an effect of the NF on expression of target genes.

In conclusion, a normalization factor was provided for dairy cattle (*RPL15*, *PPIA* and *GUSB*) and buffaloes (*YWHAZ*, *GUSB* and *GAPDH*) based on expression of the three most stable reference genes in oocytes collected from each species during winter and summer. Normalization of non-reference target genes by these reference genes was shown to be considerably different compared to normalization by less stable reference genes, further highlighting the need for careful selection of internal controls for gene expression analysis.

## Supporting Information

File S1 **Validation of the protocol used for mechanical removal of cumulus cells from oocytes.** Denuded oocytes used in molecular analyses were evaluated regarding the presence of cumulus-specific transcripts to confirm the absence of contaminating cumulus cells.
(DOC)

Figure S1 **Comparison of chemical and mechanical removal of cumulus cells.** The pictures depict oocytes that had the zona pellucida (ZP) chemically removed by treatment with 0.1% (w/v) of pronase for 5 min (A) and oocytes that were mechanically separated from cumulus cell by vortexing for 3 min at maximum speed (B). Note that no cumulus cell is attached to oocyte surface regardless of the presence of the ZP. Images were taken at 400× magnification.
(TIF)

Figure S2 **Effect of chemical and mechanical removal of cumulus cells on expression of cumulus-specific genes.** Cumulus-oocyte complexes (COCs) were denuded of cumulus cells by chemical treatment to remove the zona pellucida (without ZP) or by vortexing (with ZP). The amounts of *KITLG*, *EGFR* and *FSHR* transcripts are expressed in relation to ZP-free oocytes. Expression values were normalized by the geometric mean of *RPL15*, *PPIA* and *GUSB*. P values refer to comparisons made for each gene between oocytes with and without the ZP. Transcripts from *FSHR* were not found in both groups.
(TIF)

Figure S3 **Effect of chemical and mechanical removal of cumulus cells on expression of cumulus-specific and reference genes.** Cumulus-oocyte complexes (COCs) were denuded of cumulus cells by chemical treatment to remove the zona pellucida (without ZP) or by vortexing (with ZP). The amounts of *KITLG*, *EGFR*, *FSHR*, *RPL15*, *PPIA* and *GUSB* transcripts are expressed in relation to ZP-free oocytes. Expression values are shown without normalization by reference genes. P values refer to comparisons made for each gene between oocytes with and without the ZP. Transcripts from *FSHR* were not found in both groups.
(TIF)

Figure S4 **Comparison of expression of cumulus specific-genes between denuded oocytes and cumulus cells.** Cumulus cell pellets were collected by gentle pipetting of cumulus-oocyte complexes (COCs) and centrifugation. Oocytes that had cumulus cells partially removed by pipetting were vortexed before sampling to remove the remaining cells. The amounts of *KITLG* and *EGFR* transcripts are expressed in relation to denuded oocytes. Expression values were normalized by the geometric mean of *RPL15*, *PPIA* and *GUSB*. P values refer to comparisons made for each gene between denuded oocytes and cumulus cells.
(TIF)

Table S1 **Primers and TaqMan probes used for preamplification and real-time RT-PCR.**
(DOC)

Table S2 **Analysis of preamplification uniformity.**
(DOC)
