# Supplementary material for: Reference Gene Selection for Gene Expression Analysis of Oocytes Collected from Dairy Cattle and Buffaloes during Winter and Summer
Source: PLoS One. 2014 Mar 27;9(3):e93287. doi: 10.1371/journal.pone.0093287 (PMC3968137; doi:10.1371/journal.pone.0093287)
Supplement: File S1 — Validation of the protocol used for mechanical removal of cumulus cells from oocytes. Denuded oocytes used in molecular analyses were evaluated regarding the presence of cumulus-specific transcripts to confirm the absence of contaminating cumulus cells. (DOC) [file pone.0093287.s001.doc]

**File S1. Validation of the protocol used for mechanical removal of cumulus cells from oocytes.**

In order to validate the protocol used for oocyte sampling the following experiment was performed. KIT ligand (*KITLG*), epidermal growth factor receptor (*EGFR*) and follicle stimulating hormone receptor (*FSHR*) were used as cumulus-specific genes to evaluate their expression between zona pellucida (ZP)-free oocytes that had the ZP chemically removed and oocytes mechanically separated from cumulus cells. ZP-free oocytes were used herein as a reference group as no cumulus cells are expected to remain attached to oocytes after ZP removal.

Immature oocytes were obtained postmortem from the ovaries of cows slaughtered at a local slaughterhouse. Ovaries were transported in 0.9% saline solution at 25^o^C-30^o^C to the laboratory and follicles with diameter between 2 and 8 mm were aspirated using an 18-gauge needle attached to a 10-ml syringe. Recovered cumulus-oocyte complexes (COCs) with homogenous cytoplasm and layers of several compacted cumulus cells were selected and washed in HEPES-buffered Tissue Culture Medium (TCM-199; GIBCO BRL, Grand Island, NY, USA) supplemented with 10% fetal calf serum (GIBCO BRL), 0.2 mM sodium pyruvate, and 50 μg/ml gentamycin sulfate. A total of 200 COCs were then split into 10 pools, each one containing 20 COCs. In five of these pools oocytes had the ZP chemically removed by treatment with 0.1% (w/v) Pronase (Sigma-Aldrich Chemical Co., St. Louis, MO, USA) for approximately 5 min. In the remaining pools oocytes were mechanically separated from cumulus cells by vortexing (3 min at maximum speed). Afterwards, oocytes from both groups (with or without the ZP) were washed three times in PBS with 0.1% polyvinyl-pyrrolidone (PVP) to completely remove cumulus cells. Pooled oocytes from each group were thoroughly checked for the presence of cumulus cells under a stereomicroscope (Figure S1) and stored at -80^o^C in 0.2 ml polystyrene PCR tubes with 5 μl of PBS containing 0.1% PVP and 1 U/μl of RNase inhibitor (RNase OUT, Invitrogen, Carlsbad, CA, USA).

Transcript abundance was evaluated by real-time RT-PCR essentially as described in Material and Methods. Briefly, expression of three target genes (*KITLG*, *EGFR* and *FSHR*) and three reference genes (*RPL15*, *PPIA* and *GUSB*) was evaluated to address the issue of cumulus cell contamination. Primers for amplification of *EGFR* and *FSHR* were based on previously reported sequences [1] whereas primers for amplification of *KITLG* were a gift of Dr. José Buratini Júnior. All transcripts were amplified for 45 cycles as described in Material and Methods, except that cDNA used as template was diluted eight fold. Moreover, 1 x SYBR Green PCR Master Mix (Applied Biosystems, Foster City, CA, USA) and 200 nM of primers were used for amplification of *KITLG*, *EGFR* and *FSHR*. Positive control reactions were performed using cDNA from cumulus cells. Specificity of primers was confirmed by melt-curve analysis, electrophoresis onto 2% agarose gels and sequencing of PCR products (see Material and Methods). The expression levels of targets in relation to reference genes were compared by ANOVA (see Material and Methods) regarding the two experimental groups (with or without the ZP).

Since *KITLG*, *EGFR* and *FSHR* have been described as being expressed exclusively by cumulus cells [1–4], we expected absence of transcripts for these genes in the samples evaluated in the present experiment. This was found to be the case for *FSHR* but transcripts for *KITLG* and *EGFR* were found in all samples (Figure S2). The absence of transcripts from *FSHR* indicates that there were not contaminating cumulus cells regardless of the presence of the ZP. Furthermore, although transcripts from *KITLG* and *EGFR* were present in the samples, no difference in their expression level was found between oocytes with and without the ZP (Figure S2). No effect of experimental group was found even when expression of *KITLG*, *EGFR*, *RPL15*, *PPIA* and *GUSB* was evaluated without normalization by reference genes (Figure S3). The existence of contaminating cells should result in higher amounts of transcripts, mainly *KITLG* and *EGFR* that have been reported to be specific of cumulus cells [1–4]. Altogether, these results indicate that, if any, contamination of oocytes with RNA from cumulus cells was at similar levels between oocytes with and without the ZP.

In order to provide further evidence of absence of contaminating cumulus cells, a second experiment was performed in which expression of *KITLG* and *EGFR* was compared between denuded oocytes and cumulus cells. We expected that albeit present in denuded oocytes with the ZP, transcripts from *KITLG* and *EGFR* would be more abundant in cumulus cells. In this regard, 100 COCs were obtained as described above and split into 5 pools, each one containing 20 COCs. Cumulus cells were partially removed from pooled oocytes by gentle pipetting in PBS with 0.1% PVP followed by centrifugation of the cells at 300 x g for 5 min. After removal of the supernatant, the cell pellets were stored at -80^o^C in 0.2 ml polystyrene PCR tubes. Additionally, partially denuded oocytes corresponding to each pool were vortexed to remove the remaining cells and stored as described above. Transcript abundance was analyzed as described above using *RPL15*, *PPIA* and *GUSB* as reference genes. In agreement with our hypothesis expression of *EGFR* was 3.2-fold greater (P = 0.002) in cumulus cells compared to denuded oocytes (Figure S4). However, expression of *KITLG* was decreased (P = 0.002) in cumulus cells by 4.2 fold in comparison to denuded oocytes (Figure S4), further indicating that expression of this gene cannot be used for analysis of cumulus cell contamination in oocytes.

In conclusion, we found no difference on expression of cumulus-specific genes between ZP-free oocytes and oocytes that had cumulus cells mechanically removed, indicating that both methods are reliable for analysis of oocyte gene expression.

**References**

1. Caixeta ES, Ripamonte P, Franco MM, Junior JB, Dode MAN (2009) Effect of follicle size on mRNA expression in cumulus cells and oocytes of Bos indicus: an approach to identify marker genes for developmental competence. Reprod Fertil Dev 21: 655–664.

2. Tong G, Heng B, Ng S (2007) Cumulus-specific genes are transcriptionally silent following somatic cell nuclear transfer in a mouse model. J Zhejiang Univ Sci B 8: 533–539.

3. Kidder GM, Vanderhyden BC (2010) Bidirectional communication between oocytes and follicle cells: ensuring oocyte developmental competence. Can J Physiol Pharmacol 88: 399–413.

4. Nagyova E (2012) Regulation of cumulus expansion and hyaluronan synthesis in porcine oocyte-cumulus complexes during in vitro maturation. Endocr Regul 46: 225–235.
