# Supplementary figures and images for: Reference Gene Selection for Gene Expression Analysis of Oocytes Collected from Dairy Cattle and Buffaloes during Winter and Summer
Source: PLoS One. 2014 Mar 27;9(3):e93287. doi: 10.1371/journal.pone.0093287 (PMC3968137; doi:10.1371/journal.pone.0093287)

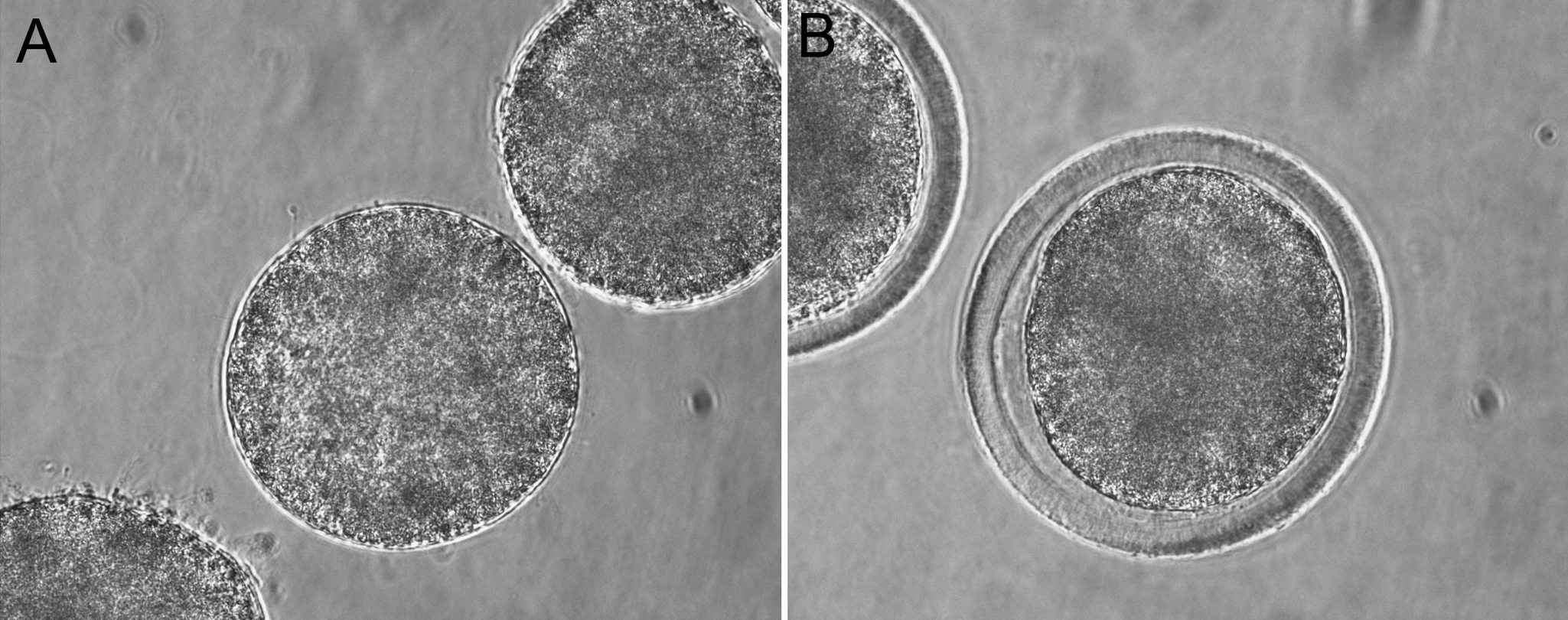

Supplement: Figure S1 — Comparison of chemical and mechanical removal of cumulus cells. The pictures depict oocytes that had the zona pellucida (ZP) chemically removed by treatment with 0.1% (w/v) of pronase for 5 min (A) and oocytes that were mechanically separated from cumulus cell by vortexing for 3 min at maximum speed (B). Note that no cumulus cell is attached to oocyte surface regardless of the presence of the ZP. Images were taken at 400× magnification. (TIF) [file pone.0093287.s002.tif]
