# Supplementary material for: Reference Gene Selection for Gene Expression Analysis of Oocytes Collected from Dairy Cattle and Buffaloes during Winter and Summer
Source: PLoS One. 2014 Mar 27;9(3):e93287. doi: 10.1371/journal.pone.0093287 (PMC3968137; doi:10.1371/journal.pone.0093287)
Supplement: Table S1 — Primers and TaqMan probes used for preamplification and real-time RT-PCR. (DOC) [file pone.0093287.s006.doc]

Table S1. Primers and TaqMan probes used for preamplification and real-time RT-PCR

| Target assay | Primer or probe | Sequence | Product |
| --- | --- | --- | --- |
| (Genbank) |  | (5’-3’) | (bp) |
| *ACTB* | ACTB-f | GGCACCCAGCACAATGAAGA | 67 |
| (NM_173979.3) | ACTB-r | GCCAATCCACACGGAGTACTT |  |
|  | ACTB-FAM | FAM-TCAAGATCATCGCGCCCCC-NFQ^1^ |  |
| *GAPDH* | GAPDH-f | CCACTCCCAACGTGTCTGTT | 84 |
| (NM_001034034.1) | GAPDH-r | GCTTCACCACCTTCTTGATCTCATC |  |
|  | GAPDH-FAM | FAM-CTTGGCAGGTTTCTCC-NFQ^1^ |  |
| *GUSB* | GUSB-f | AGAGCGAGTACGGAGCAGATG | 85 |
| (NM_001083436.1) | GUSB-r | AGCAGGCCTTTCTGGTACTCTTC |  |
|  | GUSB-FAM | FAM-TTTCACGAGGATCCACCACTGATGTTCA-BHQ1^2^ |  |
| *HIST1H2AG* | HIST1H2AG-f | CGGTGCTGGAATACCTGACA | 80 |
| (XM_868899.3) | HIST1H2AG-r | GATGATGCGGGTCTTCTTGTTG |  |
|  | HIST1H2AG-FAM | FAM-CCAGCTCTAAGATCTC-NFQ^1^ |  |
| *HPRT1* | HPRT1-f | GGGACTTGAATCACGTGTGTGT | 142 |
| (NM_001034035.1) | HRT1-r | CCACAGAACAAGAACATTGGATCA |  |
|  | HPRT1-FAM | FAM-CCTGGCGTCCCAGTGAAATCACCA-BHQ1^2^ |  |
| *HSPA1A*+*HSPA1B*^3^ | HSPA1AB-f | CGCGAAGCGGCTGATC | 83 |
| (NM_174550.1) | HSPA1AB-r | CGTTGATGACGCGGAAAGG |  |
| (NM_203322.2) | HSPA1AB-FAM | FAM-CCGGGTCTCCGAACTT-NFQ^1^ |  |
| *HSP90AA1* | HSP90AA1-f | GACACATGCCAACAGGATCTACA | 86 |
| (NM_001012670.1) | HSP90AA1-r | CGGCGCTGCTGTCATC |  |
|  | HSP90AA1-FAM | FAM-CTTGGTCTCGGTATTGAC-NFQ^1^ |  |
| *PPIA* | PPIA-f | GGTCCTGGCATCTTGTCCAT | 94 |
| (NM_178320.2) | PPIA-r | TGCCATCCAACCACTCAGTCT |  |
|  | PPIA-FAM | FAM-AATGCTGGCCCCAACACAAATGGTT-BHQ1^2^ |  |
| *RPL15* | RPL15-f | CAAACGCCCAGTTCCTAAGG | 76 |
| (NM_001077866.1) | RPL15-r | TCGAGCAAACTTGAGCTGGTT |  |
|  | RPL15-FAM | FAM-CTACGGCAAGCCTGTCCACCATGGT-BHQ1^2^ |  |
| *SDHA* | SDHA-f | GCAATAAGAGGTCGTCCGCTAA | 76 |
| (NM_174178.2) | SDHA-r | GCATCAAACTCATGGTCCACAA |  |
|  | SDHA-FAM | FAM-TTTCAGATGCGATTTCTGCGCAGTATCC-BHQ1^2^ |  |
| *TBP* | TBP-f | AACAGCCTCCCACCCTATGC | 71 |
| (NM_001075742.1) | TBP-r | AAGATAGGGATTCCAGGAGTCATG |  |
|  | TBP-FAM | FAM-CAGGGCCTGGCCTCCCCTCAG-BHQ1^2^ |  |
| *YWHAZ* | YWHAZ-f | GATATCTGCAATGATGTACTGTCTCTTTT | 106 |
| (NM_174814.2) | YWHAZ-r | CGGTAGTAGTCTCCTTTCATTTTCAA |  |
|  | YWHAZ-FAM | FAM-TGATCCCCAACGCTTCACAAGCAG-BHQ1^2^ |  |

^1^MGB probe from Applied Biosystems (NFQ = non-fluorescent quencher)

^2^Probe from Sigma-Aldrich (BHQ1 = black hole quencher 1)

^3^Termed in the current work as *HSPA1AB*
