# Supplementary material for: Reference Gene Selection for Gene Expression Analysis of Oocytes Collected from Dairy Cattle and Buffaloes during Winter and Summer
Source: PLoS One. 2014 Mar 27;9(3):e93287. doi: 10.1371/journal.pone.0093287 (PMC3968137; doi:10.1371/journal.pone.0093287)
Supplement: Table S2 — Analysis of preamplification uniformity. (DOC) [file pone.0093287.s007.doc]

Table S2. Analysis of preamplification uniformity

|  | ΔΔCt (one vs. two rounds of preamplification) | |
| --- | --- | --- |
| Genes | Bovine | Buffalo |
| *ACTB* | 0.28 ± 0.06 | 0.51 ± 0.11 |
| *GAPDH* | 0.39 ± 0.07 | 0.51 ± 0.11 |
| *GUSB* | 0.59 ± 0.55 | 0.14 ± 0.01 |
| *HIST1H2AG* | 0.23 ± 0.06 | n.a.^1^ |
| *HPRT1* | 0.16 ± 0.02 | 0.08 ± 0.02 |
| *HSPA1AB* | 2.90 ± 0.43 | 0.10 ± 0.05 |
| *HSP90AA1* | 0.07 ± 0.02 | 0.57 ± 0.08 |
| *PPIA* | 0.26 ± 0.16 | 0.11 ± 0.02 |
| *RPL15* | 0.21 ± 0.18 | 0.07 ± 0.04 |
| *SDHA* | 0.22 ± 0.09 | 0.15 ± 0.04 |
| *TBP* | 0.54 ± 0.20 | 0.31 ± 0.15 |
| *YWHAZ* | 0.27 ± 0.17 | 0.11 ± 0.07 |
| Average | 0.61 ± 0.14 | 0.24 ± 0.04 |

^1^Not analyzed
